# Biomedical Applications of Metal−Organic Frameworks for Disease Diagnosis and Drug Delivery: A Review

**DOI:** 10.3390/nano12020277

**Published:** 2022-01-16

**Authors:** Miral Al Sharabati, Rana Sabouni, Ghaleb A. Husseini

**Affiliations:** 1 Department of Chemical Engineering, American University of Sharjah, Sharjah P.O. Box 26666, United Arab Emirates; g00077296@alumni.aus.edu; 2 The Material Science and Engineering Program, College of Arts and Sciences, American University of Sharjah, Sharjah P.O. BOX 26666, United Arab Emirates

**Keywords:** metal−organic frameworks, disease diagnosis, drug delivery, theranostic agent

## Abstract

Metal−organic frameworks (MOFs) are a novel class of porous hybrid organic−inorganic materials that have attracted increasing attention over the past decade. MOFs can be used in chemical engineering, materials science, and chemistry applications. Recently, these structures have been thoroughly studied as promising platforms for biomedical applications. Due to their unique physical and chemical properties, they are regarded as promising candidates for disease diagnosis and drug delivery. Their well-defined structure, high porosity, tunable frameworks, wide range of pore shapes, ultrahigh surface area, relatively low toxicity, and easy chemical functionalization have made them the focus of extensive research. This review highlights the up-to-date progress of MOFs as potential platforms for disease diagnosis and drug delivery for a wide range of diseases such as cancer, diabetes, neurological disorders, and ocular diseases. A brief description of the synthesis methods of MOFs is first presented. Various examples of MOF-based sensors and DDSs are introduced for the different diseases. Finally, the challenges and perspectives are discussed to provide context for the future development of MOFs as efficient platforms for disease diagnosis and drug delivery systems.

## 1. Introduction

Improving health and extending the lifespan of the human population necessitate the development of therapeutic agents in the form of chemical agents and bioactive composites. Many of these composites are ideal candidates for treating acute diseases such as cancer and diabetes, as well as kidney, cardiovascular, and microbial diseases [1]. Nevertheless, significant drawbacks limit their use in biomedical applications, including poor solubility, poor body absorption, poor bioavailability, and unselective biodistribution [1]. This usually leads to damaging healthy tissues [2], burst release [3], and cardiotoxicity effects [4,5]. Utilizing a nano-based drug delivery system (DDS) can overcome these problems by increasing drug solubility and stability, controlling drug release, increasing drug bioavailability, decreasing toxic side effects, evading (bio)degradation, and providing targeted delivery to certain body parts [6,7,8,9]. The search for an efficient DDS for therapeutic agents has been a never-ending mission in several fields, including chemistry, biochemistry, and medicine, in addition to biomedical and biological engineering.

**Figure 1:**
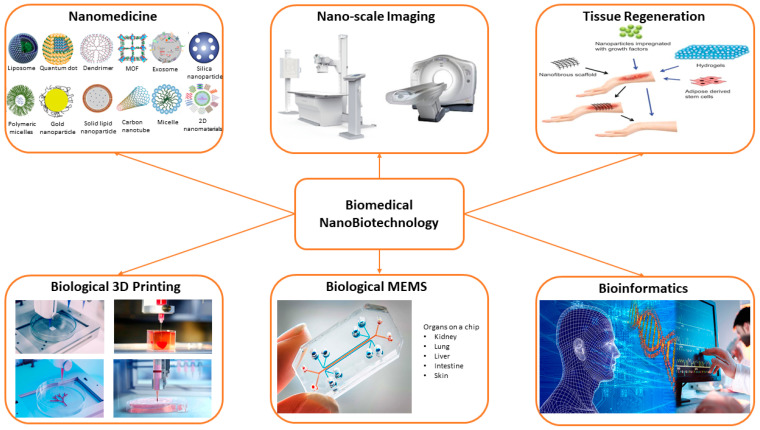
Nanotechnology applications in the biomedical field.

Nanotechnology has contributed to the development of a variety of fields, including biomedical, biological, environmental, and nutraceutical research [9,10,11] (Figure 1). Nanostructures are present in various configurations such as nanofibers, nanoparticles, nanotubes, and nanocomposites, which efficiently help in diagnosing [12] and treating different diseases [10,13,14]. In addition, these nanostructures are used as carriers or transporting agents for drugs [15], proteins [16], vaccines [17], genes [18], and enzymes [19]. Nanomedicine is a promising field that employs the information and methods of nanoscience in medical biology and disease prevention and treatment [1]. It involves the use of nano-dimensional substances such as nanorobots, nanovehicles, and nanosensors for diagnosis and delivery purposes, as well as activating materials in live cells.

Nanocarriers are colloidal systems with submicron particles or droplet sizes of less than 500 nm [20]. Thus, their movement in the human body would be easier and more accessible compared to larger particles. Nanoscale-sized particles have exceptional chemical, structural, magnetic, and biological features. In the past years, extensive investigations have been carried out on nanocarriers as they have great potential in the drug delivery field; they can encapsulate drugs or conjugate therapeutic drugs to their surface and transport them preferentially to certain tissues, where they can release their cargo [21]. Due to their large surface area-to-volume ratio, nanocarriers can change drugs’ fundamental characteristics and bioactivity [6]. Nanocarriers remain in the blood circulatory system for a long time, allowing the drugs to be released in a spatially and temporally controlled manner [22]. Small-sized nanospheres enter the tissue system, ease the drug uptake by cells, allow for effective drug delivery, and guarantee action at the targeted site. Cells can absorb nanoparticles much more than larger particles ranging in size between 1 to 10 µm [18,23].

Nanocarriers can deliver drugs via two routes, either passive delivery or self-delivery. In passive delivery, either physical encapsulation or chemical conjugation is used to combine drugs with nanostructures. Hydrophobic−hydrophobic interactions allow for the encapsulation of drugs in the inner cavity of the framework. Using targeting techniques, therapeutic agents’ release and concentration can be controlled, but in lower quantities due to the encapsulation in the inner hydrophobic environment of these nanocarriers [24]. Moreover, other non-covalent adsorption methods include hydrogen bonding, ion−ion electrostatic interactions, π–π stacking, halogen bonding, van der Waals interactions, and coordination bonding [25]. In chemical conjugation, on the other hand, there is a direct conjugation between the drugs and the nanocarrier to ease the drug delivery. It must be cleavable at the target site for good control over the triggered release. The self-delivery method is based on the ability of drugs to self-assemble. They act as building blocks in nanostructures, where their distribution and content are precisely controlled [24].

Over the past years, several shapes and sizes of nanostructures have been synthesized and used for various drug delivery systems. There are three types of nanocarriers used in drug delivery: organic, inorganic, and hybrid [3,6]. Organic nanocarriers include liposomes, polymeric micelles (PMs), solid lipid nanoparticles (SLNs), dendrimers, polymeric nanoparticles (PNPs), and protein-based nanomaterials and nanosystems. These nanoparticles are flexible, less toxic, and can conjugate various drugs and ligands for drug delivery [26]. The second type, i.e., inorganic nanocarriers, includes carbon nanotubes (CNTs), quantum dots (QDs), mesoporous silica nanoparticles (MSNs), graphene oxide (GO), gold nanoparticles (GNPs), magnetic nanoparticles (MNPs), and two-dimensional (2D) nanomaterials like metal nanosheets, graphene-based materials, MoS_2_, etc. These nanostructures have controllable features and a synergetic therapeutic effect [27]. The third type, which combines the two previous classes, is the organic/inorganic hybrid nanocarriers. Lipid−polymer hybrid, ceramic−polymer hybrid, and metal−organic frameworks (MOFs) are among the examples of this kind of nanocarrier [3,28]. This type of nanocarrier combines the advantages of both materials, which strengthens its properties [29]. Each type has its advantages and disadvantages. Table 1 presents a summary of some of the commonly used nanocarriers, along with their advantages and disadvantages.

The selection of a suitable nanocarrier type is a significant challenge of the latest studies in the biomedical field. In recent years, metal−organic framework nanocarriers have been studied for the delivery of many biomolecules. Although there have been many reviews covering several aspects of the applications of MOFs in biomedicine, limited work has combined their use as biosensors and drug delivery vehicles for more than one disease.

The current review presents the most recent progress of metal−organic frameworks as promising nanocarriers for disease diagnosis and drug delivery in the biomedical field. First, a brief introduction to MOFs and their synthesis and applications in biomedicine is provided. Then, recent diagnosis and treatment applications of MOFs for various diseases such as cancer, diabetes, and Alzheimer’s disease are demonstrated. Finally, conclusions are drawn, and challenges are summarized in anticipation that this review can pave the way for future exploration of MOFs as novel theranostic systems for biomedical applications.

## 2. Metal−Organic Frameworks (MOFs)

**Figure 2:**
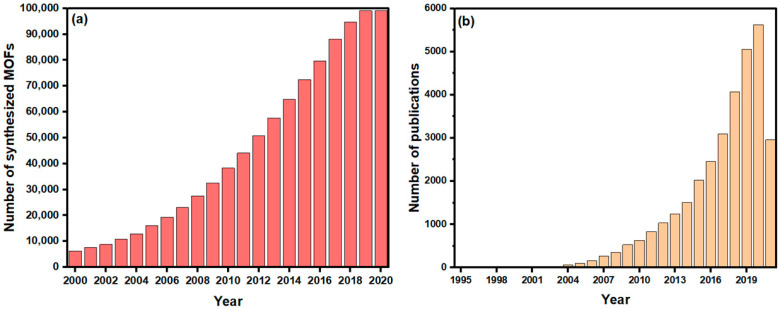
(**a**) The number of synthesized MOFs per year reported in the Cambridge Structural Database (CSD). (**b**) The number of publications per year having the keyword “Metal−organic framework(s)”, retrieved from the Web of Science on 11 July 2021.

MOFs, also known as porous coordination polymers (PCPs) [44,45], are a type of porous crystalline material that can be easily tuned, owing to the extended network structures constructed by the self-assembly of inorganic metal clusters and organic ligands [46]. Various MOF structures with high porosity frameworks can result from the flexible combination of metal ions and organic linkers, which differentiate them from other nanostructures [47]. Currently, there are about 99,075 synthesized MOF and MOF-type structures deposited into the Cambridge Structural Database (CSD), as demonstrated in Figure 2a. Due to their exceptional properties, MOFs have attracted increasing attention for multiple applications as promising and emerging porous hybrid materials, as shown from the growing number of studies investigating their applications, represented by Figure 2b. These applications include, but are not limited to, gas storage and separation [48,49], chemical separation [50], bioimaging [51,52], catalysis [53,54], water treatment [55], and energy [56,57].

Some of the unique features of MOFs include (1) high surface area and porosity (i.e., surface areas of 1000 to 10,000 m^2^/g) that increase the loading of biomolecules and the encapsulation of various types of pharmaceuticals [58], and adjustable pore sizes with diameters less than 2 nm, making them microporous structures that determine the size of the molecules that can fit in the pores [59]; (2) open architectures, which facilitate the interaction between the incorporated biomolecules and the external environment, as the substrates and products can transfer from the pores [60,61]; (3) high variety of inorganic clusters and organic ligands, which result in well-designed geometry and characteristics that can be tailored to meet their applications; (4) biodegradability, due to weak coordination bonds that are critical for the controllable release of drugs [47,62]; and (5) high crystallinity, which presents specific morphological information and definite networks, which is crucial when studying host-guest interactions [60,63]. These extraordinary properties of MOFs make them serve as one of the best candidates for disease diagnosis and drug delivery for biomedical applications.

Furthermore, in order for MOFs to be employed in the biomedical field, precise control over the particle size and morphology is required as only sufficiently small particles (<100 nm) are capable of penetrating cells [64]. An exciting new class of materials has recently emerged from scaling down MOF materials, known as nanoscale MOFs (NMOFs). They have the same ample variety of structures, compositions, and characteristics of bulk MOFs, together with the advantages of nanomaterials. The properties of nanomaterials are determined by their chemical composition, as well as their morphological properties such as shape, size, and surface characteristics. These variables influence the chemical characteristics, reactivity, energetic properties, and (photo-) catalytic activities of a substance. As the size of the materials approaches the nanoscale and the percentage of atoms at their surface becomes substantial, their characteristics change [65]. Increasing attention has been drawn to developing novel synthesis routes to generate MOF nanoparticles, although examples of NMOFs are rather uncommon. The chosen method of MOF synthesis usually determines the size of its crystals. Nevertheless, the temperature and heating rates provide extra parameters to control the nucleation process and crystal growth during MOF preparation. Several methods for NMOFs synthesis include sonochemical and microwave-assisted syntheses, surface-assisted synthesis, microemulsion synthesis, and coordination modulation [64].

**Figure 3:**
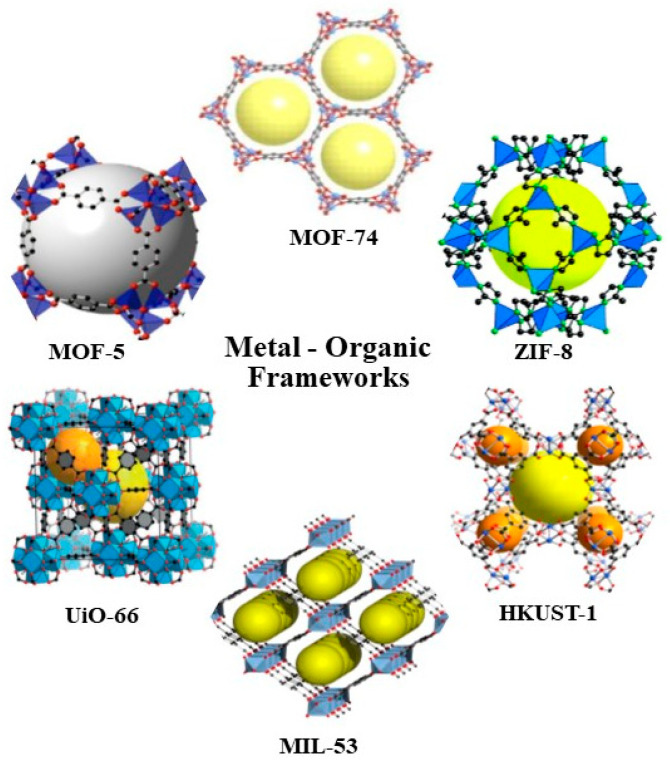
Examples of typical metal−organic frameworks [66].

Figure 3 demonstrates examples of the most investigated metal−organic frameworks [66].

### 2.1. Synthesis of MOFs

**Figure 4:**
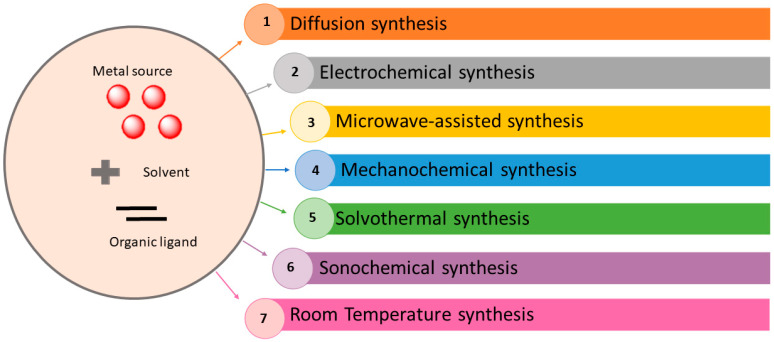
MOF synthesis methods.

Synthesis of MOFs uses experimental conditions that affect their porosity, morphology, and crystallinity [67]. Thus, it is of great importance to properly choose a synthesis method that controls the physiochemical characteristics of the acquired products. Furthermore, it is important to also consider the economic and environmental aspects, especially in large-scale synthesis. Lots of various synthetic methods can be utilized to generate MOFs, depending on the resulting frameworks and properties. As shown in Figure 4, these methods include slow diffusion [68,69], electrochemical [70,71], microwave-assisted [72,73,74], mechanochemical [75,76], hydrothermal (solvothermal) [77,78], and sonochemical [79,80,81,82] techniques.

#### 2.1.1. Diffusion Method

This method can have two approaches based on the gradual transport of different species into the interaction. One approach is solvent-liquid diffusion. Initially, two layers of different densities are formed, separated by a third solvent layer. The precipitant solvent is one of them, and the other layer surrounds the product in a solvent. At the interface, the gradual diffusion of the precipitating solvent into the dividing layer results in crystal growth. The second approach uses physical barriers to gradually diffuse the reactants, involving two vials of different sizes. In addition, gels can be applied in some cases as crystallization and diffusion media, particularly to reduce the pace of diffusion and prevent the precipitation of the bulk material. The diffusion method makes it possible to obtain single crystals that can be used for X-ray diffraction analysis as an alternative to non- or poly-crystalline products, especially if the products are insoluble [83,84,85].

#### 2.1.2. Electrochemical Method

The electrochemical synthesis of MOFs was first mentioned in 2005 by researchers at BASF [86], who synthesized HKUST-1 with the goal of eliminating anions, such as chloride, nitrate, and perchlorate through large-scale production processes utilizing MOFs [87,88]. Metal ions are continuously provided via anodic dissolution to the reaction medium as a metal source, rather than utilizing metal salts in order to react with the dissolved linker particles and a conducting salt [66,88]. The usage of protic solvents makes it possible to prevent metal deposition on the cathode, but allows for the generation of $H_{2}$ in the process. The solution to this problem is to use other solvents such as acrylonitrile, acrylic, or maleic esters. These compounds are first reduced and then slightly used to solve the issue [89,90]. Some of the advantages of this method include the synthesis process’s short time, easy crystallization, lower reaction temperatures, and easily controllable synthetic and reaction conditions throughout the synthesis process [91]. Moreover, for industrial processes, this method allows for running a continuous process and acquiring a greater solids content compared to normal batch reactions [88]. On the other hand, there are some drawbacks to using this method, as it is not well developed and difficult to handle when compared to other methods [92,93,94].

#### 2.1.3. Microwave-Assisted Method

The microwave synthesis method has been used on a large scale for the fast synthesis of nanoporous materials under hydrothermal conditions. Some examples of these materials include zeolites, manganese oxides, mesoporous molecular sieves, aluminophosphates, and, more recently, silico-aluminophosphates and other phosphates [95,96]. In this technique, the material is synthesized by microwave irradiation. The reaction time is shortened to a few hours or minutes by using microwaves that have a frequency range from 300 MHz to 300 GHz, without deteriorating the quality of the product [67]. The frequency applied affects the interactions between microwaves and electric charges of the irradiated molecules; hence, generating heat from the collisions of rotating solvent molecules. Moreover, microwave heating increases the reaction kinetics and the yield of desired products without by-products. This happens because the microwave radiates enough energy to overcome the activation energy barrier, which takes less time to complete the reaction when compared to conventional heating [97,98]. In this sort of MOF synthesis, selecting an appropriate solvent is of great importance. The suitable solvent must absorb microwave energy and transform electromagnetic energy into heat. Dielectric loss tangent is used to measure the capability of the solvent, and it has been found that the higher the dielectric loss, the more efficient the solvent [91]. The apparatus used has a pressure and temperature controller as well as tunable power outputs [66]. The reactants are simply added to a microwave-active solvent and then moved to a sealed Teflon vessel. The vessel is then put in a microwave and heated at a certain temperature for a proper time [66,95,99]. Advantages of this method include rapid crystallization [99], easy morphology control [100], high product purity [88], phase selectivity [101], and particle size reduction [88,102]. Babu et al. [74] synthesized a dual-porous metal−organic framework (MOF-205) through microwave irradiation at various time intervals. Its structural and physical characteristics were used to generate cyclic carbonates by the CO_2_-epoxide coupling reactions under solvent-free conditions. A multimode microwave reactor (KMIC-2 KW) was employed at a frequency of 2.450 GHz, with a continuously adjusted power source in the range of 0–2 kW.

#### 2.1.4. Mechanochemical Method

**Figure 5:**
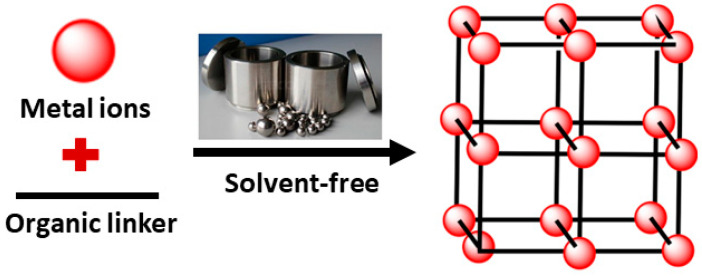
Mechanochemical synthesis of MOFs.

Mechanochemical reactions depend on the mechanical energy being directly absorbed by reagents, typically solids, in the process of milling or grinding, such as ball milling [67,91]. In this method, friction and collision between balls and reactants are the sources of energy needed to initiate the chemical reactions. Large ball collision is required to induce a chemical reaction, or else solely elastic deformations will happen. The reaction takes place quickly (10 to 60 min) at room temperature, which leads to high quantitative yields [103,104]. Solvent-free conditions are applied, which are particularly useful when organic solvents are to be avoided [104]. Therefore, it is possible to utilize insoluble metal sources that often are hard to dissolve in the solvents used in traditional syntheses of MOFs. For instance, when insoluble metal oxides are used as metal harbingers rather than salts, it is considered safer, more eco-friendly, and gives opportunities for synthesizing modern materials, as water is the only byproduct produced by metal oxides [85,103]. Nonetheless, this method is restricted to certain sorts of MOFs solely and it is hard to acquire great quantities of product [66]. Figure 5 demonstrates this type of synthesis.

#### 2.1.5. Solvothermal Method

**Figure 6:**
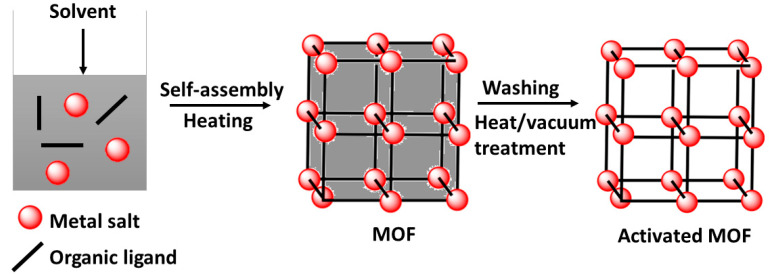
Solvothermal synthesis of MOFs.

The solvothermal method continues to be the most utilized synthesis technique amid several various synthetic techniques presented to date for the synthesis of MOFs [67]. The term solvothermal refers to the usage of any solvent in the synthesis process, while hydrothermal infers that the solvent used is water [91]. This technique includes a solvent-based reaction of metal salts with organic ligands and crystallization in a closed vessel (autoclave or sealed container), where high pressure and temperature (at or beyond a solvent’s boiling point) ease the self-assembly and crystal development (Figure 6). The choice of solvent influences both the solubility of reagents and the temperature of the reaction. Acetone, ethanol, and dimethylformamide are organic solvents that are among the most commonly used solvents in this method. During the process, traditional electric heating is the source of energy used to initiate and induce the reactions during several dozen hours. Energy can also be supplied by electrochemical, mechanochemical, and electromagnetic sources [67].

#### 2.1.6. Sonochemical Method

This type of synthesis depends on the concept of sonochemistry, where a chemical reaction occurs by applying ultrasound radiations with frequencies between 20 kHz, the upper limit of human hearing, and 10 MHz. The generation of acoustic cavitation is the mechanism behind this process [105]. Cavitation is the generation, development, and breakdown of bubbles in a liquid. As a result of the cavitation breakdown, an increase in the temperature (5000–25,000 K) and pressure (1000 atm), great heating/cooling rates, and rapid shock waves occur in the liquid around the bubble [80,106]. Sonochemical synthesis results in an incremental increase in the reaction rate, in addition to a higher yield, higher energy efficiency, and an improvement in particle synthesis. Moreover, it is eco-friendly, easy to use, can be applied at ambient temperature, and involves a substantial reduction in synthesis time compared to other traditional synthesis techniques [85,91].

Li et al. [82] utilized the ultrasonic method to synthesize a 3D metal−organic framework Cu_3_(BTC)_2_. Ultrasonic radiation was applied at an ambient temperature and atmospheric pressure for short reaction times (5–60 min), resulting in high yields (62.6–85.1%).

#### 2.1.7. Room Temperature Method

Room-temperature syntheses of MOFs are of great importance to meet the demand of sustainable chemistry, and are crucial for integrating functional compounds in water-stable MOFs [107]. This type of synthesis focuses on the direct preparation of MOFs under more sustainable conditions. MOFs are synthesized at room temperature, and thus the harmful organic solvents are replaced partly by water [108]. This method is based on the addition of an amine to a joint metal and ligand solution. This allows the precipitation to occur by the abrupt change of pH. The amine’s role is to cause the deprotonation of the ligand, leading it to react with the metal ion in the solution. Tranchemontagne et al. [109] synthesized four well-known MOFs, namely, MOF-5, MOF-74, MOF-177, and MOF-199, as well as IRMOF-0, a new isoreticular MOF, which has the same cubic topology as MOF-5, using room temperature synthesis. The study demonstrated that this type of synthesis works well for MOFs containing Cu (II) and Zn (II).

Great attention is drawn to the application of MOF in the future, as MOF’s scaling up happens through rapid reactions [110]. Multiple methods have generated various MOF materials combined with the availability of components and different process variables.

### 2.2. Biomedical Applications of MOFs

**Figure 7:**
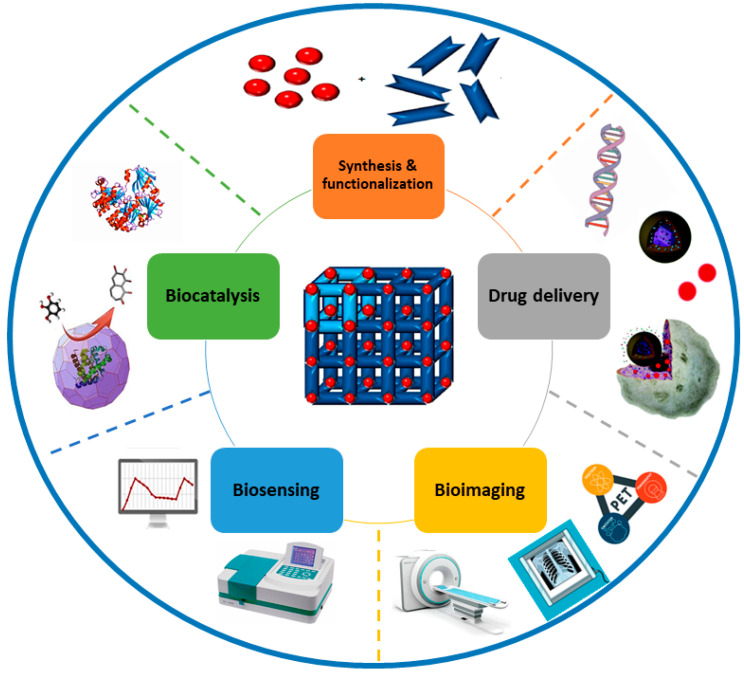
Biomedical applications of MOFs.

Due to the exceptional features of MOFs, including their high porosity, extensive surface area, large pore size, nanometer-scale size, biocompatibility, and biodegradability, MOFs have great potential in biomedical applications, including drug delivery, biosensing, bioimaging, and biocatalysis (Figure 7). MOFs can trap biomolecules into their cavities or adsorb them during synthesis [111]. They can be utilized as carriers for targeting specific body sites and for controlled release of the drugs due to their extensive surface area (1000 to 10,000 m^2^/g), high porosity, and tailorable properties. The particle size should be less than 200 nm in order for these drug carriers to freely circulate within the smallest capillaries [112]. Many sorts of functional molecules can fit within the pores because of the high porosity of MOFs and their tunable pores from microporous to mesoporous [62]. The most efficient way to entrap these molecules into MOFs is by pore encapsulation through de novo synthesis. MOF formation and substrate encapsulation happen simultaneously during the synthetic process. Thus, this approach allows for the immobilization of molecules larger than the pore size of MOFs into the cavity of MOFs. The substrate is required to be stable under synthetic conditions. This method has been commonly applied to encapsulate drugs within the MOF for intracellular delivery and consequent release [113]. For example, ZIF-8 nanospheres with a particle size of 70 nm were synthesized with the anticancer drug camptothecin encapsulated within the framework [114]. Studies on the MCF-7 breast cancer cell line showed improved cell internalization and decreased cytotoxicity.

Moreover, other examples of MOFs used to encapsulate drugs include MIL-89 (Fe) of a uniform pore size (11 Å), which was used to entrap drugs like Ibuprofen and azidothymidine triphosphate [115]. Ibuprofen was also encapsulated by some other MOFs with different pore sizes, such as HKUST-1 with a pore size of 14.67Å, MOF-2 with a pore size of 21.2 Å, and MIL-53(Fe) with a pore size of 8.6Å [116,117]. ZIF-8 with a pore size of 11.6 Å was reported to encapsulate anticancer drugs, including doxorubicin and 5-Fluorouracil [118,119].

**Figure 8:**
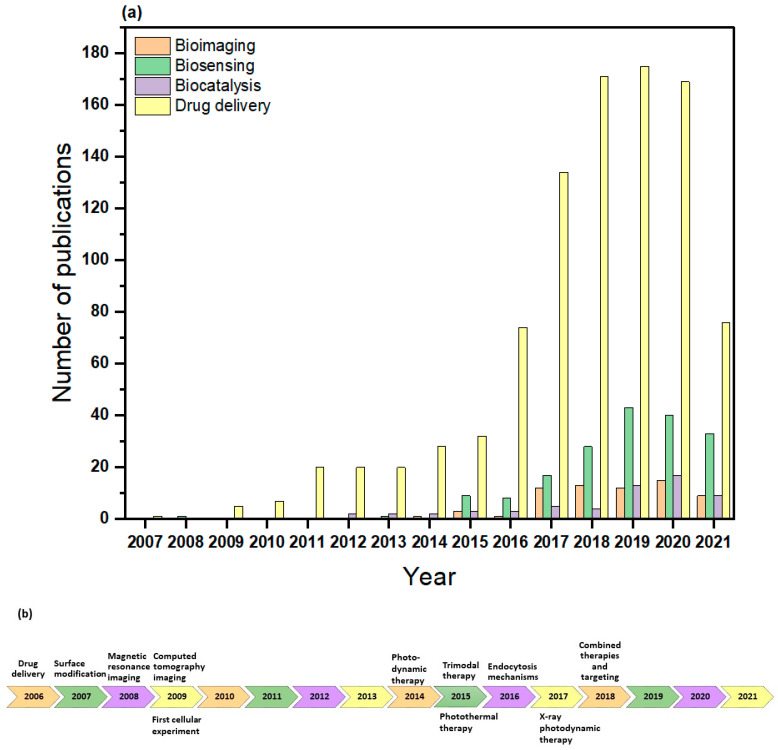
(**a**) The number of publications featuring the terms “metal−organic framework” and “bioimaging,” “biosensing,” “biocatalysis”, and “drug delivery” in their topic. Retrieved from the Web of Science on 11 July 2021. (**b**) Major milestones in the biomedical applications of MOFs [126].

In recent years, MOFs have been widely investigated in the biomedical field, particularly for drug delivery purposes, as can be seen in Figure 8a,b. In drug delivery applications, it is important that the nanocarriers have the proper design or composition, as they can effectively alter the hydrophilicity of the drugs, affect their uptake and excretion, accomplish the targeted delivery, and prevent drugs from binding to unrelated molecules [120,121,122]. In biological sensing applications, biosensors can be designed by several conjugation techniques due to the large specific surface areas of MOFs, as well as their broad range of pore shapes [123]. MOFs also act as direct alternatives to conventional enzymes for enzymatic reactions. Serving as nanozymes, they can imitate the coordination environments of the catalytic sites in natural enzymes [124]. This has wide applications in biosensing as nanozyme-based biosensors detect ions, proteins, small molecules, nucleic acids, and cancer cells [125]. Finally, in bioimaging applications, imaging contrast agents can be used to modify MOFs and develop potential targeted platforms for magnetic resonance imaging (MRI), optical molecular imaging, and X-ray computed tomography imaging (CT) [47]. Table 2 lists these applications with their different techniques, as well as examples of researched MOFs.

### 2.3. Stimuli-Responsive Therapeutic Platform Based on MOFs

The fabrication and decoration of stimuli-responsive moieties on the surfaces of metal−organic frameworks make them promising stimuli-responsive nanocarriers. This means that they can be specialized nanosized carriers that have environmentally sensitive modalities within their frameworks. Certain environmental stimuli can cause these nanocarriers to release their encapsulated drugs, and remarkably offer a new outlook for developing novel nanoformulations [141]. The nanocarriers mentioned above are particularly beneficial when the stimuli are unique to disease pathology, enabling the specific response of the nanocarrier to the pathological “triggers” [142]. Generally, these stimuli can be classified into two primary categories: internal and external [143]. Internal stimuli depend on the various physicochemical situations available at the target site, including differences in the pH, temperature, redox potential, hypoxia, and enzyme concentrations between normal and diseased cells [144,145,146]. External stimuli can attain the optimal spatio-temporal control of the drug release. Examples include magnetic field, ultrasound, electric current, light, and heat [142,144]. Table 3 lists the different types of stimuli used for stimuli-responsive drug delivery.

### 2.4. Toxicity of MOFs in Biological Systems

A great deal of attention has been directed towards the potentially toxic effects of MOFs in biological systems [147]. The broad use of MOFs might give rise to severe health threats to the living organisms exposed to these macrostructures. This has led to questioning the biocompatibility of MOFs in the biological system. Thus, the possible risks related to the applications of MOFs in these systems have to be fully understood [148].

The research in toxicological studies of MOFs is still in its early stages. Nevertheless, there are some well-known parameters that can stimulate toxicity in MOFs. The types of cross-linkers and metals, particle size, ligands, functionalized groups, and the solvent system used to synthesize the MOF are among them. In addition, the cellular uptake, biodistribution, translocation, and excretion from the body are heavily affected by the nature, amount, degradation rate, and shape of the functional groups over the surface [112].

The metal ions used in the synthesis of MOFs are in the nanoscale size and are mostly nonbiodegradable. Well-known toxic metals such as lead, arsenic, chromium, and cadmium in MOFs may cause severe health complications because of the toxicity of these MOF-forming metals. Therefore, the metals that are typically needed as nutrients for the body, such as zinc and iron, which are also the least toxic, should be used to synthesize the MOFs designed for drug delivery or other therapeutic applications.

When it comes to the organic linkers used to synthesize MOFs, carboxylates, phenolates, sulfonates, amines, and phosphonates are the most common. MOFs are expected to degrade to these constituent materials and might create severe health risks because of the characteristics of these linkers [149].

The solvents used to synthesize the MOF can also have toxic effects. They could be confined in the porous MOFs and may lead to several short-term and long-term health effects. For instance, exposure to dimethylformamide can lead to various health effects such as nausea, liver damage, vomiting, abdominal pain, alcohol intolerance, and rashes [112].

Another crucial factor that determines the MOFs’ toxicity in the biological systems is their stability. It is essential to synthesize MOFs that are stable both chemically and thermally. Components of unstable MOFs (metals, linkers, and ligands) may filtrate into cellular compartments, causing the accumulation of metallic and other species, leading to toxic effects that rely on the nature and concentration of the filtrated species. Other factors that can effectively determine the toxicity of MOFs in biological systems include the dose of the MOF, the frequency of the treatment, accumulation, and excretion patterns [150].

**Table 3:** Types of stimuli used in drug delivery.

Type of Triggers	Examples	Examples of MOFs	Drugs and applications	Remarks	Reference
Internal	pH	Hollow mesoporous silica at a zeolitic imidazolate framework (HMS@ZIF)	Doxorubicin (DOX), anticancer therapy	Engineer a system that can utilize the pH differences between the blood and the diseased cells to enable drug delivery to chosen sites.	[151,152,153,154]
		MIL-100(Fe)	Camptothecin (CPT), anticancer therapy		
		ZIF-8	D-α-Tocopherol succinate (α-TOS), antitumor therapy		
	Temperature	Zinc MOF constructed by semirigid 5-(4′-carboxyphenoxy) nicotinic acid (Zn-cpon-1)	5-fluorouracil (5-FU), anticancer therapy	Design a delivery system that will merely release the drug at temperatures beyond 37 °C.	[155,156,157,158]
		Zinc glycolate MOF (Zn-GA)	Methotrexate (MTX), anticancer treatment		
		UiO-66	5-Fu, chemophotothermal therapy		
	Redox potential (Glutathione (GSH) concentration)	Zinc-based 4,4′-dithiobisbenzoic acid MOF (MOF-Zr (DTBA))	Curcumin (CCM), anticancer therapy	Exploit the concentration gradient between normal and diseased cells, and between intracellular and extracellular environments for targeted drug delivery to certain sites.	[159,160,161,162]
		Cyclodextrin MOFs (CD-MOFs)	DOX, anticancer therapy		
		Zr-MOF	Cisplatin, anticancer therapy		
	Enzyme concentration	Porphyrinic MOF (PCN-224)	DOX, anticancer therapy	Design a system by incorporating a certain peptide sequence or chemical bond that is prone to be attacked by disease-related enzymes.	[143,163,164]
		UiO-68	CPT, anticancer therapy		
External	Ultrasound	NH_2_-Fe-BDC	DOX, anticancer therapy	Apply local sonication after the injection of encapsulated drugs for targeted delivery purposes. This enables the uniform distribution of micelles and the drug across the pathological cell.	[142,165,166]
		Fe-NDC	Calcein and DOX, anticancer therapy		
	Magnetic Field	HKUST-1	Nimesulide, anticancer treatment	After administration, the drug immobilized magnetite carrier can pile up at the targeted site under the course of an external magnetic field.	[167,168,169,170]
		PD/M-NMOF	DOX & MB, anticancer treatment		
		ZIF-8@ABFs	RhB		
	Light	o-NBA@ZIF-8	rifampicin (RFP), bacterial infection therapy	Design a light-sensitive system that goes through reverse disruption under the action of light to enable external control of drug release.	[171,172,173,174]
		UiO-AZB	5-FU, anticancer therapy		
		AuNR@MOFs	CPT, anticancer therapy		
	Heat	CP5-capped UiO-66-NH-Q	5-FU, treatment of central nervous system diseases	Apply an external heat source to raise the temperature of the cellular environment.	[155,175,176]
		CP5-capped UiO-66-NH-A	5-FU, anticancer therapy		

## 3. Applications of MOFs for Disease Diagnosis and Drug Delivery

Metal–organic frameworks (MOFs) have attracted significant interest in recent years as a promising platform for disease diagnosis, controlled drug delivery, and a combination of both (theranostic agents). This section discusses these applications in various diseases regarded as major threats to human health worldwide.

### 3.1. Cancer

Cancer is still considered a significant threat to public health worldwide, causing millions of deaths annually [177]. It is a genetic disease that involves abnormal cell growth that spreads to other parts of the body. It is anticipated that more than one-third of the population of developed countries will get cancer at some point in their lives [178]. Therefore, much effort has been made from various research fields to find innovative and efficient cancer diagnosis and treatment strategies.

MOFs have been widely studied for cancer diagnosis. Kong et al. [179] investigated a green-emission Zr (IV)-MOF (BUT-88) as a biosensing platform using a fluorescent detection technique. The MOF derivative was fabricated into a fluorescent nanoprobe, drDNA-BUT-88, which could identify dual tumor biomarkers (i.e., MUC-1 and miRNA-21) in breast cancer cells (MCF-7 cells). The probe offered improved detection precision in early cancer diagnosis, having a limit of detection (LOD) of 0.13 and 4.50 nM for miRNA-21 and MUC-1, respectively.

Another study by Sheta et al. [180] utilized a magnetic MOF-based platform (Cu-MOF-NPs) for the early diagnosis of liver cancer using the alpha-fetoprotein (AFP) quantification test. It was used as a biosensor for AFP with a detection limit of 1.18 ng mL^−1^ and a quantification limit of 3.58 ng mL^−1^ on serum samples obtained from healthy and hepatitis patients. No interference from other types of competing cancer biomarkers (interfering analytes) was noticed.

For tumor therapy, several studies have been performed to investigate MOFs as drug delivery vehicles. Herein, a few examples are presented. For instance, Kundu et al. [181] reported the utilization of a Gd^III^-based porous MOF (Gd-pDBI) for anticancer drug delivery. Gd-pDBI crystals were downsized by mechanical grinding (ca. 0.5 mm to 120 nm) to MG-Gd-pDBI. In vitro and in vivo studies demonstrated the low blood toxicity of the MOF and the high drug loading of the anticancer drug doxorubicin (DOX) (12 wt%). Release experiments using 5 wt% DOX loaded MG-Gd-pDB were performed at pH 7.4 and 5, with the latter resulting in more release (44%) than the former (22%). This DDS was found to have high water solubility, porosity, and thermal stability, as well as mild acid and base stability.

Liu et al. [182] synthesized and developed a porous Cu (II)-based MOF to encapsulate the anticancer drug 5-fluorouracil (5-FU) by a simple adsorption process. A drug loading of 37.22% was obtained by UV−VIS-Vis spectroscopy. Furthermore, it was observed that simulated cancerous tissues (at pH = 5.8 and 6.8) have (7.6–13.6%) more drug release than that in the normal tissues (pH = 7.4), indicating a pH-responsive drug release. The MTT assay confirmed the low toxicity of this DDS, in addition to its good biocompatibility and anticancer activity against cell lines A549 and HeLa.

In another study by Lei and coworkers [160], MOF-Zr (DTBA) was investigated as a redox-responsive drug carrier. Curcumin (CMM), a natural anticancer drug, was embedded within the MOF to obtain CCM@MOF-Zr (DTBA) nanoparticles, which showed a faster-releasing behavior in vitro and improved cell death in comparison to free CCM. Upon the entry of the DDS into the cancer cells, the disulfide bonds in the MOFs were cleaved by GSH, which triggers the crash of the MOFs and the release of free CCM. When the concentration of the MOF reached 400 μg mL^−1^, the cell viability was 68.4% for HeLa cells and 71.1% for MDA-MB-231 cells. In addition, the nanoparticles exhibited a higher antitumor efficacy over that of free CCM, as denoted by the in vivo studies.

MOFs have been widely investigated as theranostic systems for cancer diagnosis and therapy. For example, Zhao et al. [183] studied Fe_3_O_4_@UiO-66 core−shell composites for simultaneous magnetic resonance (MR) imaging and drug delivery. The UiO-66 shell encapsulated doxorubicin (DOX) because of the availability of the open cavities, metal sites, Zr-O clusters, and amphiphilic character, which facilitated strong coordination interactions between the Zr (IV) centers in UiO-66 and the hydroxyl groups in DOX. However, the Fe_3_O_4_ core was used as an MR contrast agent. The DDS demonstrated an excellent MR imaging ability, high drug loading capacity, continuous drug release, high stability, low cytotoxicity, and high antitumor therapeutic efficacy. In vitro and in vivo studies showed enhanced cancer cell mortality (60%), excellent tumor size inhibition, and a substantial darkening effect, making it a potential candidate for cancer diagnosis and treatment.

Moreover, a new theranostic platform consisting of Fe_3_O_4_@polyacrylic acid/Au nanoclusters/zeolitic imidazolate framework-8 nanoparticles (Fe_3_O_4_@PAA/AuNCs/ZIF-8 NPs) for the diagnosis and treatment of cancer was developed by Bian et al. [184]. Results from this study showed that these nanoparticles exhibited many advantages, including a tri-modal cancer imaging capability, ultrahigh doxorubicin (DOX) loading capacity of 1.54 mg DOX/mg NPs, dual pH-responsive controlled drug release, good biocompatibility, and easy magnetic separation. Furthermore, they demonstrated low systematic toxicity and effective chemotherapeutic efficacy in vivo.

Another study by Gao and coworkers [185] used Fe-MIL-53-NH_2_-FA-5-FAM/5-FU DDS to study its potential as a theranostic platform. Fe-MIL-53-NH_2_ was used to encapsulate the anticancer drug 5-fluorouracil (5-FU), and also served as a magnetic resonance contrast agent due to its high transverse relaxivity. Folic acid (FA) acted as the targeted reagent, while 5-carboxyfluorescein (5-FAM) was utilized for fluorescent imaging. The results showed that this nanocomposite demonstrated outstanding receptor-specific targeting, as confirmed by fluorescence imaging of FA-positive cancer cells (MGC-803 cells). The release behavior of 5-FU was found to last over 20 h and led to the DDS having better toxicity towards cancer cells as the viability of HASMC and MGC-803 cells decreased by 80%. Moreover, this DDS showed good biocompatibility, a strong cancer cell growth inhibitory effect, tumor enhanced cellular uptake, outstanding fluorescence imaging, and excellent magnetic resonance imaging capability.

### 3.2. Diabetes

Diabetes is a chronic disease that poses a significant threat to human health worldwide. It is a metabolic disease caused by insulin deficiency [186]. It causes severe long-term damage to many body organs, especially the eyes, nerves, heart, kidneys, and blood vessels [187]. Diabetes can be classified into two types, i.e., type I (TIDM) and type II (T2DM). Detection of diabetes is carried out by examining blood glucose or exhaled acetone in the body. The latter has been a more economical and noninvasive technique than the former. The acetone concentration in the breath is normally in the range of 0.3–0.9 ppm and increases to more than 1.8 ppm for diabetic patients [188]. Metal−organic frameworks have attracted increased attention in acetone detection due to their surface area and high porosities [189].

In a study conducted by Chang et al. [187], metal−organic frameworks derived ZnO@MoS_2_ nanosheets core/shell heterojunctions were used to detect acetone. Moreover, their performance was evaluated by studying the effect of acetone concentration, working temperature, and humidity. The results demonstrated the ultra-fast response to the presence of acetone, which was 9 s/17 s@ 500 ppb and 60 s/40 s @5 ppb. This is due to the ultra-fast gas diffusion rates in porous MoS_2_ nanosheets. In addition, good acetone selectivity was observed, which was explained by the considerable interaction energy and charge transfer between acetone and ZnO (MoS_2_).

Moreover, Gutiérrez et al. [190] worked on transforming non-luminescent MOFs to highly luminescent frameworks that display a high selectivity to acetone and can be used to manufacture fluorometric sensors to diagnose and monitor diabetes. The transformation was done by exposing the non-luminescent MOF (OX-1 (Zn-BDC) MOF) to a silver salt solution for a short period, leading to OX-2 (Ag-BDC) MOF. The latter was found to have an intense green luminescence with an emission quantum yield reaching 22% in powder form. Quenching this green emission in the presence of acetone makes the MOF a promising candidate to be used in breathalyzers for diabetes diagnosis.

Other researchers investigated the detection of diabetes by monitoring the glucose level of human blood. Diabetic patients have an excessive glucose content in their bloodstream. While many detection methods exist, electrochemical enzyme-free sensors have stood out as the most attractive. This is due to their lower detection limits, better stability, and lower environment reliance [191].

Wang et al. [186] investigated metal−organic framework-derived nickel/cobalt-based nanohybrids to detect glucose in the blood. The pyrolysis of a bimetallic (Ni and Co) metal−organic framework (NiCo-MOF) was carried out at 800 °C under a nitrogen atmosphere to prepare nickel/cobalt (NiCo) alloy nanoparticles coated with graphitized carbon. Since it is composed of non-noble metal nanomaterials and highly conductive carbon materials, it acts as an active and selective catalyst for glucose detection in non-enzyme sensors. Human blood samples were used to measure the serum glucose levels using these NiCo/C-modified electrodes. The linear detection of the sensor was in the range of 0.05 μM–4.38 mM, and had a limit of 0.2 μM under optimal voltage conditions (0.50 V). Moreover, good repeatability and long-lasting stability were observed.

In one study, Wei et al. [192] designed a cobalt metal−organic framework modified carbon cloth/paper (Co-MOF/CC/Paper) hybrid non-enzyme button-sensor to detect glucose. This portable, easy-to-use electrochemical analytical chip increased the specific area and catalytic sites compared to a conventional plane electrode. Glucose levels were measured in the serum, saliva, and urine and showed high durability, selectivity, stability, and excellent robustness.

#### 3.2.1. Anti-Diabetic Agents

The pancreas produces insulin, which is a hormone that controls the concentration of glucose in the blood. So far, the only effective treatment method has been direct insulin injection for insulin-resistant (IR) patients. The development of oral insulin delivery methods has been a breakthrough in diabetes treatment. They are essential to decrease the pain and discomfort that patients suffer from due to being routinely injected with insulin. Because of their instability in the stomach’s acidic environment (Ph = 1.5–3.5), a limited number of MOFs are suitable for insulin encapsulation and oral delivery [193].

The use of a crystalline zirconium-based mesoporous MOF, NU-1000, to encapsulate insulin was performed by Chen et al. [193]. A high loading efficiency of ~40 wt% was obtained in only 30 min due to the rapid encapsulation of insulin that easily spreads across the structure and interacts with the pore surface under mild conditions. When imitating stomach conditions, it was found that NU-1000 capsules prevented the degradation of insulin in the presence of stomach acid and the digestive enzyme pepsin. On the other hand, simulating bloodstream conditions led to the slow degradation of the MOF and the release of the encapsulated insulin, which maintained most of its activity.

Another study by Zhou et al. [194] designed a modified iron-based MOF nanoparticle (MIL-100) for oral insulin delivery. The MIL-100 nanoparticles were modified with sodium dodecyl sulfate (SDS) and embedded into biodegradable microspheres to enhance resistance to the stomach acid environment. Ins@MIL100/SDS@MS showed a high loading efficiency of 35% and facilitated insulin penetration across Caco-2 monolayers. Under acidic conditions, the microspheres prevented the rapid degradation of the MOF NPs and released insulin-loaded MOF NPs under imitated bloodstream conditions. Studying their effect on diabetic rats, these nanocomposite vehicles decreased blood glucose levels with a relative pharmacological availability of 7.8%, as the plasma insulin levels were considerably improved over 6 h after their oral administration compared to the oral administration of free insulin or Ins@MIL100/SDS.

Furthermore, Zhang et al. [195] developed a novel glucose-responsive delivery system (ZIF@Ins&GOx) by loading insulin and glucose oxidase (GOx) into pH-sensitive ZIF-8 nanocrystals. GOx oxidizes glucose into gluconic acid after getting into the cavities of ZIF-8, leading to a reduction in local pH. After this, under acidic conditions, the degradation of the MOF nanocrystals trigger the release of insulin. The rigid structure of the MOF protected the biological activity of insulin and promoted its encapsulation, as confirmed by in vitro studies. In vivo experiments showed no risk of hypoglycemia in type I diabetes mice, as a single subcutaneous injection of these nanocrystals stabilized blood glucose levels for an extended period (i.e., 72 h).

#### 3.2.2. Wound Healing

Chronic nonhealing wounds remain a major problem for diabetic patients, a significant challenge for physicians, and contribute to increasing healthcare expenditures [196,197,198]. Specifically, diabetic foot ulcers (DFUs) are responsible for at least 73,000 nontraumatic lower-limb amputations, and inflict a significant cost burden of more than $9 billion on public and private payers on top of other costs related to diabetes itself [196,197].

Metal−organic frameworks paired with copper ions have been involved in some wound-healing-related processes. In a recent study, wound healing in diabetic mice was investigated using copper metal−organic framework nanoparticles (HKUST-1 NPs) by Xiao et al. [196]. For a slow release of copper ions, the MOF was incorporated within an antioxidant thermoresponsive citrate-based hydrogel poly-(polyethyleneglycol citrate-co-N-isopropylacrylamide (PPCN), and both in vivo and in vitro studies were performed. Wound closure rates and wound blood perfusion were evaluated in vivo using the wound diabetic mouse model. The nanoparticles were safe from degradation and copper ions were gradually released. The study results indicated a major reduction in cytotoxicity and apoptosis caused by the release of copper ions, while wound closure rates and dermal cell migration were substantially improved. During the in vivo wound healing study, the hydrogel composite caused collagen deposition, angiogenesis, and re-epithelialization in diabetic mice.

Additionally, another study from the same group (Xiao et al. [197]) reported the modification of copper-based MOF for diabetic wound healing. During the synthesis of MOF, folic acid was added to produce folic-acid-modified HKUST-1 (F-HKUST-1). The addition of folic acid to the MOF stabilized it by enhancing its hydrophobicity and decreasing the BET surface area. This resulted in the slow release of copper ions, reducing cytotoxicity and improving in vitro cell migration (~25%) and in vivo diabetic wound healing. The in vivo studies had similar results to those reported in the previous study. On the other hand, HKUST-1 copper MOF was studied as a releasing vehicle of nitric oxide (NO) to treat diabetic wounds by Zhang et al. [198]. NO has been used as a gas medicine to treat diabetic wounds, but challenges still arise when it comes to controlling its release behavior in the affected area. In this study, NO was loaded within HKUST-1 by the electrospinning method; then, the loaded particles were embedded into the core layer of the coaxial nanofiber. The controllable release of NO was achieved with an average release rate of 1.74 nmol L^−1^ h^−1^ over 14 days. Copper ions were also released from the degradable MOF. Together with NO, endothelial cell growth and enhanced collagen deposition, angiogenesis, and the elimination of inflammation in the wounds accelerated wound healing. This resulted in the diabetic wound being completely healed within two weeks.

Recently, Li et al. [199] used a cobalt-based metal−organic framework (ZIF-67) loaded with a pro-angiogenic drug (dimethyloxalylglycine (DMOG)) to accelerate diabetic wound healing. The drug-loaded ZIF-67 nanoparticles were embedded into micro-patterned PLLA/gelatin nanofibrous scaffolds. A high loading capacity of 359.12 mg g^−1^ was obtained, and the nanoparticles were well incorporated within the patterned scaffold. A continuous release of DMOG and Co ions from the scaffold was observed for over 15 days. This resulted in facilitating the migration, proliferation, and tube formation of the endothelial cells. Furthermore, in vivo experiments showed that these scaffolds substantially improved collagen deposition and angiogenesis, and eliminated inflammation at the wound sites.

In addition, MOFs can be used as the precursors in the synthesis of single-atom catalysts (SACs), which are a series of advanced nanomaterials currently being used for biomedical applications. MOF-derived single-atom catalysts can be applied for wound healing. Xu et al. [200] used a zinc-based MOF (ZIF-8)-derived carbon nanomaterial that contains atomically dispersed zinc atoms as a single-atom peroxidase mimic. Using an in vivo infected wound model, it promoted highly effective wound healing without substantial toxicity to multiple tissues and organs, suggesting that it demonstrates both a high therapeutic effect and biosafety for wound healing.

### 3.3. Neurological Diseases

Neurological diseases are brain and spinal cord disorders denoted by a gradual deterioration of neuronal structures and/or functions [201]. They are classified into three groups, i.e., neurotraumatic, neurodegenerative, and neuropsychiatric, presented in Table 4, along with some of their examples. These disorders are affected by several unknown causes and factors, and show various symptoms. Most of them are associated with the initiation of oxidative stress and dysregulation of the inflammatory network. However, neurodegeneration can be caused by inherited genetic abnormalities, environmental and endogenous factors related to aging, and immune system problems.

Recently, research has determined glutathione (GSH) as a factor related to various neurological diseases such as autism, schizophrenia, and Alzheimer’s disease [202]. It is an antioxidant that helps prevent damage to significant cellular components due to reactive oxygen species, like free radicals, in the body. It consists of cysteine, glycine, and glutamic acid, and has sulfhydryl as its characteristic group. Therefore, accurate measurement of GSH levels in the serum would help detect and diagnose these disorders. The normal concentration of GSH in cells is in the range of 0.5 to 10 mM, and a decrease in that concentration is a potential early diagnostic biomarker. Zhu et al. [203] investigated a MOF-based fluorescence probe, namely Eu^3+^/Cu^2+^@ UiO-67-bpydc, to detect GSH in serum samples with a high sensitivity. The interaction between the thiol and Cu^2+^ ions enabled the coordination between biomolecules that consist of sulfhydryl groups (GSH) with Cu^2+^ chelated on the probe. Study results indicated that an increase in the probe’s fluorescence intensity was related to the GSH detection, which was below the normal level in the serum and cell.

Another study by Chen et al. [202] proposed a dual-sensing platform for biothiols (GSH) along with Hg^2+^, which counter-proof the existence of one another with a high specificity and can be used for the early diagnosis of neurodegenerative disorders. A zwitterionic 3D MOF of {[Cu(Cdcbp)(bipy)]·4H_2_O}_n_ was loaded with FAM-labeled T-rich P-DNA to form the sensing platform of P-DNA@MOF. A fluorescence “off−on−off” process was used for the consecutive detection of Hg^2+^ and biothiols.

Alzheimer’s disease (AD) is a fatal neurodegenerative disease and represents the most common type of dementia around the world [204]. It is identified by the gradual loss of cognitive capacity, memory loss, and functional impairment [205]. As life expectancy increases, the prevalence of this disorder increases, affecting people over the age of 65. Some ions may be the main cause of AD as it was found that the presence of metal ions (Cu^2+^, Fe^3+^, Al^3+^, and Zn^2+^) in the brain of AD patients was higher than the normal range by 3–7 times [205]. Many MOF fluorescent biosensors have been developed to detect these ions for AD diagnosis. Some examples of these MOFs are presented in Table 5.

In addition to their application in AD diagnosis, MOFs have also contributed to treating the disease. For MRI and targeted drug delivery, Zhao et al. [210] synthesized a MOF, namely Fe-MIL88B-NH2-NOTA-DMK6240, making it a promising theranostic platform. They based their research on the tau pathological hallmark, which states that increases in tau aggregation and phosphorylation are associated with worsening cognitive impairment. Tau, a protein that acts as a stabilizer of microtubules in neurons, results in AD development once it is hyperphosphorylated. Methylene blue was loaded in the MOF pores to inhibit tau aggregation and decompose tau fibrils. In addition, it was used as a magnetic resonance contrast agent. The surface was modified with DMK6240 to enhance hyperphosphorylated tau targeting, leading to the formation of an advanced DDS.

The other pathological hallmark of AD, studied by Wang et al. [211], is abnormal amyloid-β peptide (Aβ) aggregation in the brain. Porphyrinic Zr metal−organic framework (MOF) PCN-224 nanoparticles were used for NIR-light-induced efficient inhibition of Aβ into a β-sheet-rich structure in order to suppress Aβ aggregation to treat AD. The results of this study indicated that photoactivated PCN-224 nanoparticles had the ability to reduce the aggregative activity of Aβ and the cytotoxicity for PC12 cells.

### 3.4. Ocular Diseases

Ocular diseases are the leading causes of vision impairment, and can deteriorate vision to the point of blindness [212]. Some of the most common ocular diseases include glaucoma, macular degeneration, and blepharitis. Ocular drug delivery is an extremely challenging task, as only 5% of the administered drug actually reaches the intraocular tissues [213]. Metal−organic frameworks have been appraised as promising nanocarriers for ocular drug delivery. Table 6 presents two of these diseases, along with some examples of MOF-based DDSs used for their treatment.

### 3.5. Lung Diseases

Lung disease refers to a group of disorders that affect the lungs and prevent them from working properly. The most common lung diseases include asthma, chronic obstructive pulmonary disease (COPD), fibrosis, acute lung injury (ALI), and many other breathing problems. Asthma often causes chest tightness, coughing, recurrent wheezing, and breath shortness [218]. Hydrogen sulfide (H_2_S) is regarded as an early detection biomarker for asthma, as its levels in the lung would be substantially decreased in asthma patients. MOFs have been reported as a sensing platform for the detection of H_2_S in biological samples. For example, a recent study showed that fluorescent MOF composites in diluted serum samples spiked with H_2_S could be used to diagnose asthma [127]. In this study, a new MOF composite, namely Eu^3+^/Ag^+^@UiO-66- (COOH)_2_, referred to as EAUC, was synthesized. To produce this platform, Ag^+^ and H_2_S were chosen as the inputs, while the output was the fluorescent signal (I_615_) of EUC. EAUC demonstrated a high selectivity, real-time in situ detection of H_2_S, and great sensitivity with a limit of detection of 23.53 μM. MTT assay studies in PC12 cells demonstrated the low toxicity of the MOF, as well as its favorable biocompatibility, making it a suitable candidate for H_2_S detection in vivo.

Another interesting study for H_2_S detection based on the fluorescence “turn-on” strategy was presented by Zhu and coworkers [219]. The developed bimetallic MOFs (Fe_x_Al_1-x_-MIL) showed extremely efficient fluorescence quenching caused by substituting a small amount of Al^3+^ by Fe^3+^ due to the strong ligand to metal charge transfer between π-conjugated BDC-NH_2_ ligands and unpaired electrons in Fe^3+^. The results of this study demonstrated that Fe_0.05_Al_0.95_-MIL could be utilized for H_2_S detection as a fluorescence augmentation was noticed, with a good linear relationship between the H_2_S concentration (0–38.46 μM) and fluorescence intensity. Specifically, S^2−^capturing Fe^3+^ promoted the partial degradation and consequent release of BDC-NH_2_ ligands, which were identified to be real fluorophores that participate in fluorescence improvement.

In addition to H_2_S, the high level of eosinophilia in the peripheral blood and tissues of asthma patients is also considered a detection biomarker. Eosinophil infiltration significantly affects the inflammatory response of asthma. Wang et al. [218] investigated a fluorescent Zn (II)-based MOF for the treatment of childhood asthma. The MOF induced eosinophils apoptosis and reduced the level of bcl-2 gene. For in vivo detection, the enzyme-linked immunosorbent assay (ELISA) method was used to detect the release of the MBP and EDN from eosinophils in a children asthma model. The results showed a reduction in the inflammatory response, making this MOF a promising candidate for the treatment of children’s asthma.

Lung tissue has a high surface area, low metabolic activity, and short transfer route into the bloodstream; thus, drug delivery by inhalation can be performed for localized targeting, has fast onset times for therapeutic action, and induces fewer side effects [220]. Hence, using inhalation to directly treat lung diseases has been a promising method. A study presented by Li et al. [220] used γ-cyclodextrin MOF particles (CD-MOFs) for targeted drug delivery by dry powder inhalers to treat ALI. The study involves loading paeonol (PAE) as the model drug into inhalable sizes of CD-MOF particles. A high drug release of 90% was achieved in a phosphate buffer at pH = 7.4. In addition, in vivo experiments showed fast absorption by lung tissues (4.0 min) and a high absolute bioavailability (71%) of PAE when rats inhaled PAE-CD-MOF dry powder inhaler, which was improved compared to oral administration. The in vitro cellular permeability studies showed a high improvement in PAE permeability (~5 folds) after being encapsulated into CD-MOF compared to pure PAE.

Moreover, a recent study by Strzempek et al. [221] investigated loaded Fe-MIL-100 with theophylline, a methylxanthine-based drug, as a DDS for the treatment of asthma and COPD. The results indicated a high drug loading of 32% and prolonged release of 46%. Furthermore, the biotoxicity tests indicated that even at high concentrations (100 and 500 µg mL^−1^) of Fe-MIL-100, a slight effect was observed on the viability of cells. These results confirmed that this MOF could be utilized safely as a carrier for inhalable treatments.

### 3.6. Bacterial Infections

Bacterial infections are among the most severe physiological conditions threatening public health [222]. Therefore, researchers have examined numerous treatments to overcome irreparable damage over the past few decades [223]. In general, oral or intravenous antibiotics have been used to treat infections that need long-term drug administration. Nevertheless, antibiotic treatment becomes very difficult after the long-term abuse of antibiotics due to pathogens’ growing drug resistance, leading to another major issue for the existing healthcare system [224]. Thus, developing advanced antibacterial systems and therapies to overcome this problem is of high importance. Recently, metal−organic frameworks have been investigated as alternatives to antibiotics as they are suitable for the formation of nanosystems with chemical antibacterial properties. For example, Wyszogrodzka et al. [225] evaluated Fe-MIL-101- NH_2_ as a theranostic carrier of isoniazid antibiotic for tuberculosis therapy. MOF particles were loaded with 12% isoniazid, and showed sustained drug release and acted as an effective MRI contrast agent. Another study by Esfahanian et al. [226] investigated Fe_3_O_4_@PAA@ZIF-8 for ciprofloxacin (CIP) delivery. *Staphylococcus aureus* (*S. aureus*) and *Escherichia coli* (*E. coli*) bacteria were used to test their antibacterial activity. A high drug loading capacity of 93% was reported, and about 73% of the drug was released within 2 days. Table 7 presents other studies that investigated MOFs as promising antibacterial agents.

### 3.7. Viral Infections

Viruses are microscopic organisms that cannot replicate by themselves and must infiltrate a host cell of a living organism [231]. The outbreak of many viruses has highly challenged public health. These include, but are not limited to, the Ebola virus, Zika virus, human immunodeficiency virus (HIV), hepatitis A virus (HAV), and the ongoing most recent coronavirus (SARS-CoV-2) [232]. It is crucial to detect viruses as early as possible to prevent and treat the associated diseases such as the Ebola virus disease (EVD), Zika virus disease, acquired immune deficiency syndrome (AIDS), COVID-19, influenza, and rabies [231]. Lately, MOF-based sensing technology has been progressively applied for virus detection. Herein, the most recent studies investigating MOF-based platforms for detecting various viruses are discussed.

Qiu et al. [233] studied a 3D Cu(II)-based MOF of {[Cu(Cmdcp)(phen)(H_2_O)]_2_·9H_2_O}_n_ to develop a fluorescent sensor to simultaneously detect Ebolavirus conserved RNA sequences and ebolavirus-encoded microRNA-like (miRNA-like) fragment. MOF was loaded with P-DNA and used for synchronous fluorescence analysis. It showed a high sensitivity for these two target RNAs with detection limits at the picomolar level (60 pmol/L for and 206 pmol/L) and a high selectivity.

In another work, Qin and coworkers [234] reported a 3D dysprosium (Dy) MOF {[Dy (Cmdcp)(H_2_O)_3_](NO_3_)·2H_2_O}*_n_* for the fluorescence detection of Ebolavirus RNA sequences. It was shown that the MOF platform could non-covalently interact with probe ss-DNA with a high selectivity and sensitivity. A detection limit of 160 pM was recorded, suggesting that the formed system can be a promising fluorescence sensing platform.

Zhang et al. [235] used an ultrasensitive switchable electrochemiluminescence (ECL) RNA sensing platform based on Fe-MIL-88 MOFs and metal−organic gel (MOG) for Zika virus detection. A DNA probe containing an apurinic/apyrimidinic (AP) site was used to connect them, leading to a turn-off signal. A broad detection range from 0.3 nM to 3 μM was shown, with a detection limit as low as 0.1 nM. In addition, the sensor demonstrated great specificity, stability, and acceptable real sample detection capability. Xie et al. [236] reported the simultaneous detection of Dengue and Zika virus RNA sequences by a 3D Cu-based MOF [Cu(Dcbcp)(bpe)]_n_. This MOF can form electrostatic, π stacking, and/or hydrogen bonding interactions with two different fluorophore-labeled DNA probes to form two P-DNA systems. Using a single detection method, the detection limits were 332 and 192 pM, while they were 184 and 121 pM with a synchronous fluorescence detection method. No cross-reaction between the two probes was observed for synchronous detection. The detection efficiency was enhanced when using synchronous fluorescence analysis by increasing the detection limits.

Moreover, another study focusing on the selective detection of hepatitis A virus using molecularly imprinted polymers (MIPs) based on a magnetically fluorescent MOF (MIL-101-NH_2_) [237] was conducted by Wang et al. [237]. The MOF served as both a fluorescence signal generator and imprinting carrier in this study. An excellent selectivity and high detection sensitivity were reported for the constructed MIP sensor. The sensor had a detection limit of 3 pM and was capable of detecting viruses within 15 min while maintaining its high selectivity.

Yang and coworkers [238] investigated a fluorescence molecularly imprinted sensor, MIL-101@SiO_2_ NPs, for Japanese encephalitis virus (JEV) detection in serum samples. Polyethylene glycol (PEG) was utilized as a blocking agent to increase the recognition rate of the template virus. The sensor exhibited a broad range of detection, 50 pmol L^−1^ to 1400 pmol L^−1^, within 20 min, and a low detection limit (13 pmol L^−1^). The surface passivation technology resulted in an improved selectivity to the template virus (imprinting factor = 4.3).

Several studies reported using MOFs as therapeutic agents for the treatment of viral infections. Marcos-Almaraz et al. [239] used an iron(III) trimesate MIL-100(Fe) nanoMOF to co-encapsulate two active triphosphorylated nucleoside reverse transcriptase inhibitors (NRTIs), azidothymidine triphosphate (AZT-Tp) and lamivudine triphosphate (3TC-Tp), to improve anti-HIV therapies. The drug loading capacity reached 9.6 wt% with an equivalent AZT/STC ratio. Freeze-drying was used to store the drug-loaded particles for up to 2 months, maintaining the same physicochemical properties. In vitro studies showed antiretroviral activity of the drug-loaded nanoMOFs on monocyte-derived macrophages experimentally infected with HIV. When treating HIV patients, they might be beneficial in reducing NRTI medications, targeting HIV reservoirs, or serving as protecting microbicide.

### 3.8. Miscellaneous Diseases

MOFs have been used as diagnostic or therapeutic platforms for other diseases; examples are listed in Table 8.

## 4. Challenges and Future Directions

Although MOFs have exceptional properties that promote their use as platforms for disease diagnosis and drug delivery, several challenges still exist in this field. There are limited studies that have been reported on the biological applications of these materials. Degradability, blood circulation half-life, stability, and selectivity are significant characteristics of the MOFs to operate in the body, and the final controlled releasing/imaging/sensing efficiency differs with these various characteristics. To date, there has not been any systematic comparison on the efficacy of MOFs, which leads to a lack of understanding of their characteristics for biological applications [47]. Moreover, the synthesis of stable and monodisperse formulations of MOFs is still a critical problem due to their degradable character. Surface modification suffers from the same problem, as results initially seem promising. Still, there must be an evaluation of the surface modification, as well as the final stealth of the resulting surface-engineered nanoparticles [243]. Furthermore, additional attention should be directed towards the biocompatibility and toxicology of MOFs. There is very limited and insufficient information on the in vivo toxicity, pharmacokinetics, and biodistribution of different novel MOFs, which are essential for the preclinical biocompatible evaluations of these developing materials. So far, several in vitro toxicity studies have been performed on various cell lines, so it is very hard to compare the results [62]. In addition, an extensive comprehension of the reaction and metabolism mechanism is needed to enhance the performance of MOFs before clinical implementation [47]. MOFs will be among the most promising materials in the biomedical field in the future, and hopefully, this work will pave the way towards more advanced studies in this field.

## 5. Conclusions

Due to their outstanding chemical and physical characteristics, MOFs have been the focus of extensive research for various applications. Specifically, exploring MOFs as sensing platforms and drug delivery systems in biomedical applications has attracted increased attention in the past few years. In this review, a brief description of MOFs was presented, along with their different synthesis methods, highlighting the advantages and disadvantages of each method. A variety of diseases have been discussed by introducing the recent MOF-based platforms that have been used as potential candidates for their diagnosis and treatment. These diseases include cancer, diabetes, neurological diseases, ocular diseases, lung diseases, bacterial and viral infections, etc. Although significant progress has been made using MOFs for biomedical applications, further improvement is needed before MOFs can become viable diagnostic and therapeutic options.

## Figures and Tables

**Table 1:** Nanocarriers for targeted drug delivery.

Nanocarriers	Structures	Advantages	Disadvantages	References
Liposomes (organic)	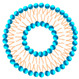	Biocompatibility, biodegradability, lower toxicity, prevention of drug degradation, reduction in side effects when encapsulating therapeutic agents, targeted drug delivery to diseased tissues, capable of delivering both hydrophilic and hydrophobic drugs, cost-effective formulations of expensive drugs	Instability, low encapsulation efficiency, insufficient drug loading, poor controlled drug release, shorter circulation times in the blood, poor storage stability, weak chemical and physical protection of sensitive drugs	[30,31,32,33,34,35,36,37,38]
Polymeric Micelles (PMs) (organic)	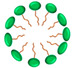	Biostability, high drug loading capacity, easy to functionalize their surface, long circulation times in blood, reduction in side effects, targeted and controlled drug release	Immature drug release, prone to aggregation and opsonization in the bloodstream	[24,39,40,41]
Carbon Nanotubes (CNTs) (inorganic)	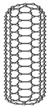	Dynamic strength, unique physiochemical properties, high drug entanglement, intrinsic stability, good biocompatibility, structural flexibility, suitable surface functionalization, low cytotoxicity	Potential asbestos-like effects, low drug delivery capacity within the CNTs	[3,6,11,27]
Quantum Dots (QDs) (inorganic)	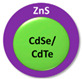	Distinctive electronic properties, luminescence features, high light stability, continuous absorption, narrow emission bandwidth, versatile surface chemistry	Derivatization and ligands required	[6,42,43]

**Table 2:** Biomedical applications of MOFs with examples.

Biomedical Application	Technique	Description	Reference
Drug Delivery	Encapsulation of therapeutic cargoes	Includes: The two-step encapsulation route, where the size of molecules are < than MOFs pores. In-situ encapsulation routes, where the size of molecules are > than MOFs pores such as proteins	[127,128]
	Conjugations of therapeutic agents to the linkers	The attachment of therapeutic agents to ligands via orthogonal conjugation. An example would be the incorporation of amino groups into the framework by doping the terephthalic acid ligand with 2-aminoterephthalic acid during the growth of Fe-MIL-101 MOFs.	[129]
	Therapeutic agents as linkers	Direct incorporation of therapeutics as building blocks for MOF synthesis such as the synthesis of MOFs with porphyrin derivative-based linkers for photodynamic therapy (PDT). An example would be the synthesis of Hf–porphyrin nanoscale DBP–UiO MOF.	[130]
Bioimaging	Magnetic Resonance Imaging (MRI)	A diagnostic method dependent on the nuclear magnetic resonance of particles in the body that produces computerized images by analyzing the absorption and transmission of high-frequency radio waves. Mn, Fe, and Gd-based MOFs are some examples of potential candidates for this application.	[47,131,132]
	X-ray Computed Tomography Imaging (CT)	A 3-D visualization of internal structures based on the mitigation of X-rays that produces a sequence of tomographic images at various orientations. Photoactive (UiO-PDT) MOF is an example of a CT contrast agent	[133]
	Optical Imaging (OI)	Light illumination is used to achieve real-time visualization with minimally invasive operations. An example is the incorporation of indocyanine green (ICG) into MIL-100(Fe) with a high loading capacity of 40 wt%, which is coated with a layer of hyaluronic acid (HA) for tumor-targeting.	[134]
	Positron Emission Tomography (PET)	High-resolution 3D images of metabolic processes in the body are given by recording the positrons emission from radioactive materials piling up at the target organs or tissues. Zr-based MOFs were used for PET imaging.	[135]
Biosensing	Nucleic acid sensing	Nucleic acid levels facilitate disease diagnosis and observe biological systems since DNAs and RNAs are crucial for physiological control. The incorporation of the triplex-forming oligonucleotide with H_2_dtoaCu MOFs to detect HIV DNA sequences is one example of this method.	[136]
	Intracellular molecules sensing	Many diseases can be reflected by the presence of small biomolecules such as glucose and metal ions in the tissue. R-UiO based bio-MOF was used as a phosphorescence/fluorescence dual-emissive platform for intracellular oxygen ratio measurement.	[137]
	Intracellular pH sensing	The reflection of the alternations of physiological environments. F-UiO MOFs were developed for real-time intracellular pH sensing via conjugating fluorescein isothiocyanate with the Zr-based MOFs	[138]
	Intracellular temperature sensing	The detection of the temperature difference between normal and diseased cells. Thermosensitive near-infrared LnMOF is an example.	[139]
Biocatalysis	Biomimetic catalysis	Certain MOFs have very effective catalysis as well as very low toxicity features. This makes them appropriate candidates for disease diagnosis and immunoassay. Nanometric MIL-100 implemented the intrinsic peroxidase-like catalytic activity for ascorbic acid colorimetric sensing.	[140]

**Table 4:** Classification of neurological diseases [201].

Neurological Disorders	Examples
Neurotraumatic diseases	Stroke, spinal cord injury, and traumatic brain injury
Neurodegenerative diseases	Alzheimer’s, Parkinsons, and Huntingtons
Neuropsychiatric diseases	Autism, depression, and hyperactivity

**Table 5:** MOFs used for the detection of metal ions related to Alzheimer’s disease.

Metal Ion	MOF	Remarks	Reference
Zn^2+^	Cd_2_(L^1^)(DMF)_2_(H2O)_2_	Zinc ions were selectively fluorescent detected over mixed metal ions in a methanol solution.	[206]
Cu^2+^	[Me_2_NH_2_][Eu(ox)_2_(H_2_O)]·3H_2_O	A 3D Eu-MOF was decomposed upon the exchange of copper ions with a cationic guest molecule, leading to luminescent quenching.	[207]
Al^3+^	Eu(L^4^)(OAc)(DMA)	The attachment of aluminum ions on the probe’s surface reduces the energy transfer between Eu^3+^ and the ligand, resulting in luminescent quenching.	[208]
Fe^3+^	BUT-14 BUT-15	BUT-15 showed a better sensing ability as its pyridine N donors donate their long-pair electrons to Fe^3+^ ions.	[209]

**Table 6:** Ocular diseases with their MOF-based DDSs.

Ocular Disease	Description	MOF Nanocarrier	Drug	Loading Capacity	Remarks	Reference
Glaucoma	It affects the anterior segment of the eye and is characterized by an increased pressure in the eyes that damages the optical nerve. It is the second leading cause of irreversible blindness worldwide. Known as “the silent thief of sight” as it has no symptoms or signs	UiO-67 MIL-100 (Fe)	Brimonidine	50–60 wt%	Cytotoxicity assays showed the high biocompatibility of the MOFs.	[214]
		NH_2_-MIL-88(Fe)		121.3 μg mg^−1^	In vivo studies showed that the nanocarriers stayed on the preocular surface for a long period (4 h), resulting in an increase in drug bioavailability.	[215]
		Zr-based UiO-67 and polyurethane MOF (UiO-67@ PU)		58.4 mg g^−1^	The MOF-based polymer nanocomposite showed a prolonged drug release (14 days).	[216]
Photoreceptor Degeneration	It is one of the most refractory oculopathy in the world. Severe cases suffer from vision loss.	Nanoscale zirconium-porphyrin MOF (NPMOF)	Methylprednisolone (MPS)	97.3 wt%	NPMOF demonstrated excellent in vivo biocompatibility and low biotoxicity. After one intraocular injection, faster photoreceptor regeneration of the retina was achieved with better visual function.	[217]

**Table 7:** Examples of MOF therapeutic platforms for antibacterial applications.

MOF Platform	Bacteria	Drug	Remarks	Reference
Fe-MIL-101-NH_2_	*Mycobacterium tuberculosis*	Isoniazid	A theranostic agent for drug delivery and imaging properties. The drug dissolution showed continuous drug release inside the L929 fibroblast cells.	[225]
MIL-100 (Fe) nanoparticles (NPs)	*Bacteria membrane*	3-azido-d-alanine (D-AzAla)	Fast degradation and accumulation after intravenous injection. Selective integration of d-AzAla into the cell walls of bacteria.	[222]
MOF-53 (Fe) nanoparticles (NPs)	*Staphylococcus aureus*	vancomycin (Van)	Efficient drug loading capacity of 20 wt% and high antibacterial efficiency of 99.3%. Excellent stability under acidic conditions. Excellent biocompatibility.	[227]
Fe_3_O_4_@PAA@ZIF-8	*Escherichia coli* *Staphylococcus aureus*	ciprofloxacin (CIP)	High loading capacity (93%) and drug release (73%). Inhibition of bacterial growth.	[226]
Ag-doped magnetic microporous γ-Fe_2_O_3_@SiO_2_@ZIF-8-Ag (FSZ-Ag)	*Escherichia coli* *Staphylococcus aureus*	-	Release of 80% of Ag in the solutions, leading to the suppression of bacteria growth.	[223]
hydrogel@ Cu-MOF	*Escherichia coli* *Staphylococcus aureus*	-	Excellent antibacterial activity at 2 mg mL^−1^ due to the large surface area to volume ratio and the antibacterial property of copper.	[228]
Ag-doped carbonized ZIF nanocomposites(C-Zn/Ag)	*Escherichia coli* *Staphylococcus aureus*	-	Fast and safe wound sterilization and can be an alternative to antibiotics. 100% bactericidal ratio for highly concentrated bacteria (10^7^ CFU/mL) within 10 min.	[224]
L-arginine and glucose oxidase encapsulated Cu-MOFs (L-Arg/GO*x*@CuBDC)	*Escherichia coli* *Staphylococcus aureus*	-	Coencapsulation of glucose oxidase (GO*x*) and l-arginine (l-Arg). High antibacterial efficiency of ≥97% at very low doses.	[229]
Silver-based metal−organic framework embellished with graphene-oxide (GO-Ag-MOF)	*Escherichia coli* *Bacillus subtilis*	-	Outstanding antibacterial activity Elimination of 95% of live bacteria.	[230]

**Table 8:** MOFs used as therapeutic agents for other types of diseases.

Disease	Description	MOF platform	Remarks	Reference
Chronic kidney disease (CKD)	It is defined by the gradual loss of kidney function over time.	DIBc NMOF	Significant improvement and recovery of glomerular basement membrane thickening and wrinkling, mesangial matrix expansion, meningeal hypercellularity, and intra-cytoplasmic hyaline droplets after 8 weeks of treating male Wistar rats with DIBc.	[240]
Chronic Toxoplasmosis	It is caused by infection with the Toxoplasma gondii parasite	Curcumin@Fe-MOF and UiO-66-NH_2_	Treatment of infected rats with these nanocomposites resulted in a significant decrease in the number of brain cysts (parasite load).	[241]
Hemorrhagic Shock	It occurs due to large amounts of blood loss which leads to reduced cardiac output and tissue perfusion	ZIF-8 encapsulating free hemoglobin (ZIF-8@Hb)	Better biocompatibility, less protein adsorption, and macrophage uptake. Extension of blood circulation and reduction of nonspecific distribution in normal organs. Significant extension of survival time was observed in treated mice.	[242]
